# The effects of passive and active administration of heroin, and associated conditioned stimuli, on consolidation of object memory

**DOI:** 10.1038/s41598-022-24585-9

**Published:** 2022-11-27

**Authors:** Travis Francis, Michael Wolter, Francesco Leri

**Affiliations:** Department of Psychology and Neuroscience Specialization, University of Guelph, 50 Stone Road East, Guelph, ON N1G 1Y4 Canada

**Keywords:** Classical conditioning, Consolidation

## Abstract

Mode of administration (i.e., active vs passive) could influence the modulatory action that drugs of abuse exert on memory consolidation. Similarly, drug conditioned stimuli modulate memory consolidation and, therefore, acquisition and extinction of this conditioned response could also be influenced by mode of drug administration. Exploring these questions in male Sprague–Dawley rats, Study 1 assessed memory modulation by post-training 0, 0.3 and 1 mg/kg heroin injected subcutaneously in operant chambers (i.e., drug conditioned context). Study 2 asked a similar question but in rats trained to self-administer 0.05 mg/kg/infusion heroin intravenously, as well as in rats that received identical amounts of intravenous heroin but passively, using a yoked design. The period of heroin exposure was followed by repeated drug-free confinement in the conditioned context, and by sessions during which responses on the active lever had no scheduled consequences. Study 2 also included a cue-induced reinstatement session during which lever responses reactivated a light cue previously paired with intravenous heroin infusions. The post-training effects of injected/self-administered/yoked heroin, extinction and reinstatement sessions on memory consolidation were tested using the object location memory task. It was found that post-sample heroin enhanced memory in injected and yoked, but not self-administering, rats. However, post-sample exposure to the heroin cues (i.e., context or/and light cue) modulated memory equally in all groups. Taken together, these data support the conclusion that mode of administration impacts the cognitive consequences of exposure to drugs but not of environmental stimuli linked to their reinforcing effects.

## Introduction

Drugs of abuse influence behaviour in part through their ability to enhance memory consolidation; a process by which recently acquired memories are stabilized over time^1,2^. In fact, when passively administered immediately after training (i.e., post-training method^3^) heroin, cocaine, amphetamine, alcohol, nicotine, and caffeine enhance acquisition of a variety of tasks, in a variety of species^4–10^. These observations have implications for addiction because drugs that enhance the consolidation of actions performed prior to, or during, drug intoxication have the potential to increase the probability that these behaviours will be repeated in the future^11^.

Interestingly, environmental stimuli associated with the effects of drugs (i.e., conditioned stimuli; CSs) also acquire the ability to enhance memory consolidation^9,10^. Hence, using the spontaneous object recognition memory task, we found that drug-free rats exposed immediately post-training to drug-paired CSs displayed enhanced object memory when tested 72 h later^9,10^. This is remarkable because it is well established that drug paired CSs play multiple roles in addictive behaviors such as maintaining drug intake^12,13^, modulating tolerance^14^, and precipitating relapse^15,16^. As recovery programs often focus on decreasing the intensity of responses to drug CSs by cue-exposure^17^, it is valuable to establish whether the memory effects of CSs can also be affected by extinction procedures.

Furthermore, when considering the relevance of the memory effects of drugs and their CSs to addictive behaviours, it becomes apparent that drugs are usually self-administered, rather than passively received, and there is significant evidence in animals that whether a subject self-administers or passively receives a drug differentially impact brain systems involved in memory consolidation^18–23^. For example, dopamine (DA) activity in the nucleus accumbens (NAc) plays a critical role in modulating memory consolidation^24,25^ and DA levels in this region are differentially affected by mode of administration^19^. Moreover, DA is involved in cue learning in both humans and animals^26,27^ and it is currently unknown whether mode of drug administration would also impact the development of conditioned memory modulation by drug CSs. In other words, in a typical self-administration situation, subjects would be exposed passively and actively to various contextual and discrete drug cues, and it is not clear whether different CSs would be equally capable of becoming memory modulators.

The first objective of the current studies was to explore whether mode of administration could impact the ability of heroin to modulate memory consolidation. Therefore, in Study 1, rats received passive subcutaneous (SC) injections of different doses of heroin in a specific context. The same context (i.e., operant chambers) was used in Study 2 to answer the question of whether self-administered intravenous (IV) heroin would have a similar effect on memory. However, because of many pharmacokinetic differences between SC and IV heroin, Study 2 also included a yoked group that received identical amounts of IV heroin, but passively^19,28,29^.

The second objective of both studies was to examine different aspects of conditioned memory modulation. Therefore, Study 1 explored the effects of repeated, drug-free exposure to the heroin-paired context on its ability to modulate memory consolidation. Similarly, Study 2 explored extinction of conditioned memory modulation by a heroin-paired context accompanied by simultaneous extinction of lever pressing. Study 2 also assessed possible memory modulation by a non-extinguished discrete light CS that would be expected to reinstate lever-pressing^15,30^.

Because memory tasks based on the innate tendency of rats to explore novel stimuli are sensitive to memory enhancement by drugs of abuse and their CSs^10^, this study employed an object location memory task^31^. Therefore, using the post-training method to selectively modulate consolidation^3^, animals were first exposed to two identical objects in two locations (i.e., sample phase), and then are immediately exposed to heroin (SC, self-administered IV or yoked IV) and/or to the heroin paired CSs. After a 72 h retention period, rats were tested for their ability to discriminate between familiar and novel locations of sample objects. Because previous studies in our laboratory indicated that at, this retention interval, drug-naïve rats cannot discriminate between familiar and novel objects^9,10^, the observation of significant discrimination at choice is interpreted as enhanced consolidation of object memory.

## Materials and methods

### Subjects

Eighty-eight male Sprague–Dawley rats (Charles River, QC) weighing between 250 and 300 g at the beginning of the experiments were individually housed in standard rat cages (polycarbonate; 50.5 × 48.5 × 20 cm) with standard environmental enrichment. Upon arrival, rats were given 1 week of acclimatization to the facility and were maintained on a 12-h reverse light/dark schedule (lights off 7:00 A.M., on 7:00 P.M.). All behavioral testing was conducted during the dark period. Rats had access to 25 g per day of standard rat chow, and water ad libitum in their home cages. All procedures followed the guidelines of the Canadian Council on Animal Care and ARRIVE and were approved by the University of Guelph Animal Care Committee.

### Surgery

Rats in Study 2 were surgically implanted with IV silastic catheters (Fisher Scientific, Whitby, ON) in the right jugular vein under general anesthesia induced by isoflurane (4% induction, 2% maintenance). Meloxicam (5 mg/kg SC, Ontario Veterinary College, Guelph, ON) was administered approximately 30 min before and 24 h after surgery. Rats were given atropine sulfate (10 mg/kg SC, Ontario Veterinary College, Guelph, ON) prior to surgery and received a small injection of Lidocaine (2.0%–0.05 ml) at the sites of incision. Depocillin (300,000 IU, 0.1 ml/rat IM, Ontario Veterinary College, Guelph, ON) was administered immediately following surgery. The catheter was secured to the vein with silk sutures and was passed SC to the back of the rat, approximately 3 cm posterior from the front shoulder blades where it exited into a connector (a modified 22-gauge cannula; Plastics One, Roanoke, VA) secured to surgical mesh with dental cement. A plastic blocker was placed over the opening of the cannula when not in use. Catheters were flushed daily with saline and every second day with 0.1 ml of a saline–heparin solution (15 IU/ml Heparin, Ontario Veterinary College, Guelph, ON). Rats were given at least 7 days to recover after surgery before behavioural testing began.

### Apparatus

#### Operant chambers

Twenty Plexiglas operant chambers (model ENV-008CT, Med Associates, Georgia, VT) were each enclosed in larger sound-attenuating plywood cabinets (model ENV-018 M, Med Associates). Each operant chamber contained a house light (28 V), and two levers, one retractable (active) and one stationary (inactive), located 10 cm apart and 8 cm above the floor. The active lever entered and remained extended during the entire duration of all sessions, and all responses were recorded. In addition, presses on this lever activated a white light (28 V) located 3 cm above the lever that served as a discrete CS that was paired with heroin delivery in Study 2. The inactive lever served to control for non-specific lever responding, presses on this lever had no consequence but were recorded. Presses on the active lever also activated infusion pumps (Razel Scientific Instruments, Stanford, CT) positioned outside the sound-attenuating cabinets that were used to deliver IV heroin infusions in Study 2.

#### Object location memory

The object location memory task assesses the ability of rats to discriminate between familiar and novel locations of objects placed in an open field^31^. The apparatus consisted of an open box (70 cm × 70 cm × 60 cm) made of white corrugated plastic. The floor was black and two walls opposite from each other were covered with two distinct patterns. Objects used varied in height, size, and texture. An overhead camera was used to record object exploration while in the apparatus.

### Procedures

#### Study 1

##### Conditioning

In this study, conditioning involved pairing the effects of injected heroin (unconditioned stimulus; US) with an operant chamber (context CS), in the presence of levers and a discrete light stimulus also employed in Study 2 (see below).

Rats were randomly assigned to one of three doses of heroin (0, 0.3, or 1 mg/kg; *n* = 16 per group). For each of the 6 conditioning sessions, rats were transported from their home colony to the testing room, injected, and immediately confined in the operant chambers for 1 h. The number of US-CS pairings was established based on our previous place conditioning studies with heroin^10,32^. Each session began with activation of the house light, entry of the retractable lever, and activation of the discrete light stimulus above the lever for 30 s. After this interval, lever presses activated the discrete light stimulus for 5 s, but no infusions were delivered. Responses on the stationary lever were also recorded and had no consequences.

##### Extinction

Each session lasted 1 h, using the above conditioning parameters, but all rats received SC injections of vehicle prior to confinement in the chambers. The total number of extinction sessions was established based on our previous place conditioning studies with heroin^10,32^.

##### Object location memory tests

Each test included a sample and a choice phase. During the sample phase, two identical objects were placed approximately 5 cm from the walls and 15 cm from each other in adjacent corners of the apparatus, and rats were allowed to explore both objects for a total of 180 s, until 25 s of total object exploration was reached, or whichever came first. Object exploration was defined as the nose pointed directly at the object within 2 cm and/or touching the object with the nose. Following a 72-h retention period, rats were tested in the choice phase, during which the apparatus contained the same two sample objects, but one object was moved to a new location. The choice phase lasted 2 min, and the time spent exploring each object was recorded. Time spent investigating objects were scored by an experimenter blind to experimental group allocations.

**Figure 1:**
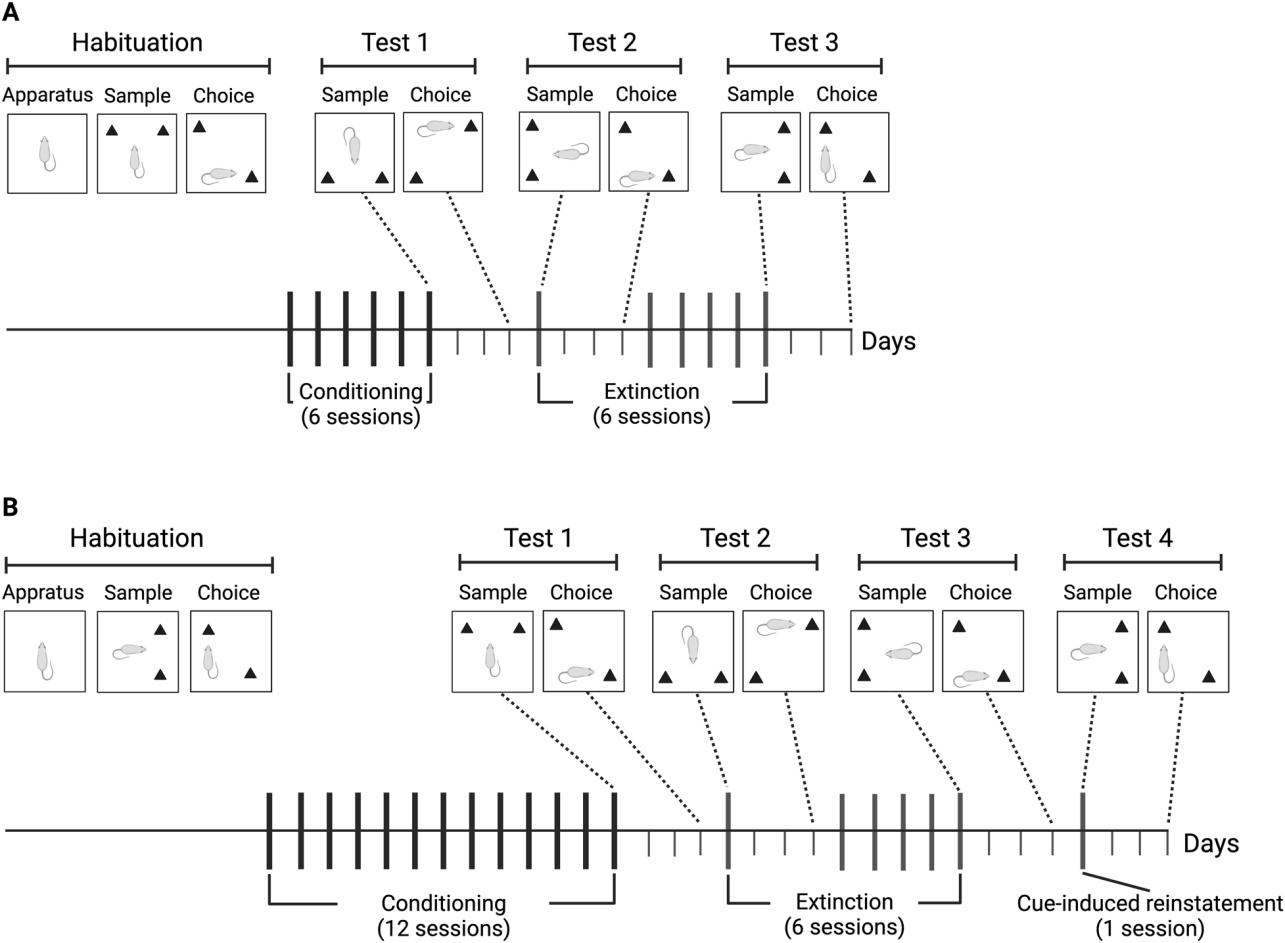
(**A**) experimental design employed in Study 1 showing the relationship between conditioning and extinction sessions to the tests of object location memory. (**B**) experimental design employed in Study 2 showing the relationship between conditioning, extinction, and reinstatement sessions to the tests of object location memory.

Figure 1A illustrates how the object location memory task was employed to explore the effects of conditioning and extinction sessions on the consolidation of object location memory. Study 1 investigated whether conditioning and extinction sessions experienced immediately after the sample phase would improve discrimination between familiar and novel object locations assessed 72 h later during the choice phase.

For habituation, rats were placed in the object location apparatus for 10 min and in 4 subsequent days, exposed to the task as described. The 6th conditioning session was selected to assess the effect of heroin as well as the context CS on consolidation because it was reasoned that animals would have learned the CS-US association by then. Therefore, for Test 1, immediately following the sample phase, rats were injected with 0, 0.3 or 1 mg/kg heroin and placed in the chambers for 1 h. At the conclusion of the session, they were transferred to their home colony where they remained undisturbed for 72 h. After this retention period, they were transported back to the testing room, placed in the open field, and assessed for object location memory during the choice phase.

The same animals were retested to assess the effects of exposure to the context CS in the absence of heroin, before and after extinction. Thus, Tests 2 and 3 were conducted using the same procedures as Test 1, but in conjunction with the 1st and 6th extinction session (Fig. 1A).

It is important to note that during all sample and choice phases of all tests, rats were drug-free. As well, on each test, rats were exposed to new, never-before-seen objects, and the locations of the moved object were counterbalanced for each rat and for each test.

#### Study 2

##### Self-administration/yoked conditioning

Following recovery from surgery, rats were randomly assigned to self-administration (SA; n = 20) or yoked (Y; n = 20) groups. The SA group was trained to actively self-administer 0.05 mg/kg/inf heroin on a fixed ratio 1 (FR1; one lever press per infusion). Rats in the Y group were individually linked to rats in the SA group by connecting the infusion pumps and the discrete light CS. For each of the 12, 3 h conditioning sessions, SA and Y rats were transported from their home colony to the testing room, placed in the operant chambers and attached to the infusion lines. Each session began with activation of the house light, entry of the retractable lever, and illumination of the discrete light CS above the active lever for 30 s. If a SA rat responded on the active lever during this first period, it received a 150 µl infusion of heroin. Responses made by Y rats on both active and inactive levers were without consequences. Similarly, subsequent presses on the active lever by SA rats led to heroin infusions and simultaneous illumination of the discrete light CS for 5 s, but responses by Y rats were without consequences. Importantly, each rat in the Y group received a heroin infusion and activation of the light CS each time its paired SA rat received one. This procedure ensured that rats in the two groups received the same pattern and number of pairings between the context CS and heroin infusions, and between heroin infusions and the discrete light CS above the active lever.

##### Extinction

For these 1 h long sessions, the same parameters used during self-administration/yoked conditioning were employed, but responses on the active lever by SA rats did not lead to heroin infusions nor did they result in activation of the discrete light CS for both SA and Y rats. In other words, the chamber was no longer paired with heroin infusions (as in Study 1), and operant responding was no longer followed by heroin infusions or by the activation of the discrete light CS. Extinction was conducted over 6 sessions because prior work indicated that this number of sessions is generally sufficient to produce a significant decrease in lever pressing^33^.

##### Reinstatement by the light CS

For this final 1 h session, the same parameters used during extinction were employed, but active lever presses by the SA rats now resulted in activation of the discrete light CS for both them and their yoked counterpart. This procedure was used because previous work indicated that introduction of a response-contingent heroin-paired CS after extinction of lever pressing reliably reinstates operant responding^33^.

##### Object location memory tests

Using the same logic and approach of Study 1, the object location memory task was employed to explore the effects of self-administration/yoked conditioning, extinction, and reinstatement sessions, on the consolidation of object location memory (Fig. 1B).

### Drugs

Heroin (Diacetylmorphine hydrochloride, Toronto Research Chemicals, Toronto, ON) was dissolved in 0.9% physiological saline and injected SC at a volume of 1.0 ml/kg in Study 1 or self-administered/yoked IV at a dose of 0.05 mg/kg/infusion and a volume of 150 µl/infusion in Study 2. The range of SC heroin doses was selected based on observations that both 0.3 and 1 mg/kg reliably produce a conditioned place preference^32^, and that post-training administration of these doses enhance acquisition in a similar object memory task^10^. The intravenous dose was selected based on previous self-administration studies in our laboratory^33–35^.

### Data analysis

Analysis of object location memory involved the calculation of a discrimination ratio (DR) in the first minute of the choice phase using the formula: [(time exploring object in novel location – time exploring object in familiar location)/total exploration time]^36^. A score of 0 indicated equal exploration of both objects, while a positive score indicated more time spent investigating the object in the novel location. A sample DR was also calculated using an if/then scenario: (if “the right object is in a novel location” during the choice phase, then [(right object exploration – left object exploration)/total exploration of both objects]. A minimum exploration time was not used in these calculations. Sample vs choice DRs were compared using paired t-test within group, and the alpha level was adjusted using the Bonferroni correction. Total object exploration for each sample and choice was calculated to rule out possible non-specific drug effects on object exploration. Because this variable was never significantly different between groups, data are not shown, and statistical analyses not reported.

Three-factor mixed repeated measures ANOVA were used to analyze lever responses. In cases where the assumption of sphericity was violated, the Greenhouse–Geisser (GG) corrected P value was used. In case of significant interactions, individual mean differences were identified by multiple simple comparisons using the Bonferroni correction. Analyses were performed using Statistical Package for the Social Sciences (V28 for Mac, SPSS Inc., IBM). The threshold for significant difference was < 0.05. Lever pressing in Study 1 was recorded, but because it was not associated with delivery of heroin, data are not shown.

## Results

### Study 1

**Figure 2:**
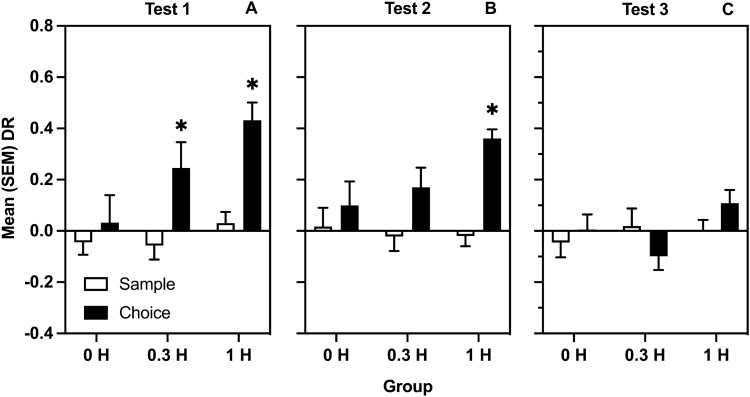
Effects of injected heroin and exposure to context CS on object location memory. (**A**) mean (SEM) DRs from sample and choice phases of 0, 0.3, and 1 mg/kg groups on the test of memory modulation by the last conditioning session. (**B**) mean (SEM) DRs from sample and choice phases on the test of memory modulation by the first extinction session. (**C**) mean (SEM) DRs from sample and choice phases on the test of memory modulation by the last extinction session. The * indicates a significant difference between phases.

Figure 2A represents mean (SEM) sample and choice DRs on Test 1, performed in conjunction with the last conditioning session. T-tests between sample and choice DRs were significant for 0.3, [*t*(15) = − 3.11, *p* = 0.007] and 1 mg/kg [*t*(15) =  − 4.42, *p* < 0.001] heroin groups only.

Figure 2 also represents mean (SEM) sample and choice DRs for Test 2 (Panel B, performed in conjunction with the first extinction session) and Test 3 (Panel C, performed in conjunction with the last extinction session). T-tests on Test 2 sample and choice DRs was significant [*t*(15) = − 6.20, *p* < 0.001] only in the group conditioned with 1 mg/kg heroin. On Test 3, sample and choice DRs were not different in any group [*p* = 0.49*; p* = 0.209*; p* = 0.12].

### Study 2

At the conclusion of self-administration/yoked conditioning, catheter patency was confirmed by connecting a piece of tubing to a 1 ml syringe, attaching it to the cannula, and slowly pulling back on the syringe. Catheters were considered patent if blood could be drawn. This verification excluded 4 rats from data analysis leading to SA group n = 18 and Y group n = 18.

**Figure 3:**
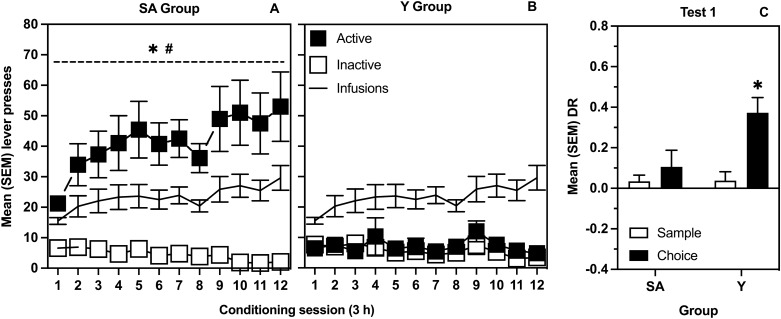
Effects of self-administered and yoked heroin on lever responses and object location memory. (**A**) mean (SEM) active and inactive lever presses for rats in the self-administration group (SA; n = 18). (**B**) mean (SEM) active and inactive lever presses for rats in the yoked group (Y; n = 18). The * indicates a significant difference between levers. The # indicates a significant difference in responding between SA and Y groups on the active lever. (**C**) mean (SEM) DRs from sample and choice phases of SA and Y groups on the test of memory modulation by the last conditioning session. The * indicates a significant difference between phases.

Figure 3 represents mean (SEM) responses on the active and inactive levers across all 12 self-administration sessions for SA (Panel A) and Y (Panel B) groups, as well as the number of infusions. The ANOVA revealed significant Lever by Group [*F*(1, 34) = 33.5, *p* < 0.001] and Session by Lever [*F*(3.93, 133.6 GG corrected) = 2.72, *p* = 0.033] interaction effects as well as a significant main effect of Lever [*F*(1, 34) = 39.1, *p* < 0.001]. Multiple comparisons indicated that responses on the active lever were significantly greater than on the inactive lever as well as in comparison to active lever responses of the Y group across all sessions, and that there was a significant increase in active lever responses from Session 1 to Session 12 in the SA group only. The t-test comparing number of infusions delivered during session 1 and session 12 was significant, [*t*(17) = − 3.73, *p* = 0.002].

Figure 3C represents mean (SEM) sample and choice DRs for Test 1, performed in conjunction with the last conditioning session. The t-test comparing sample and choice DRs was significant only in the Y group [*t*(17) = − 4.67, *p* < 0.001]; SA group, *p* = 0.42].

**Figure 4:**
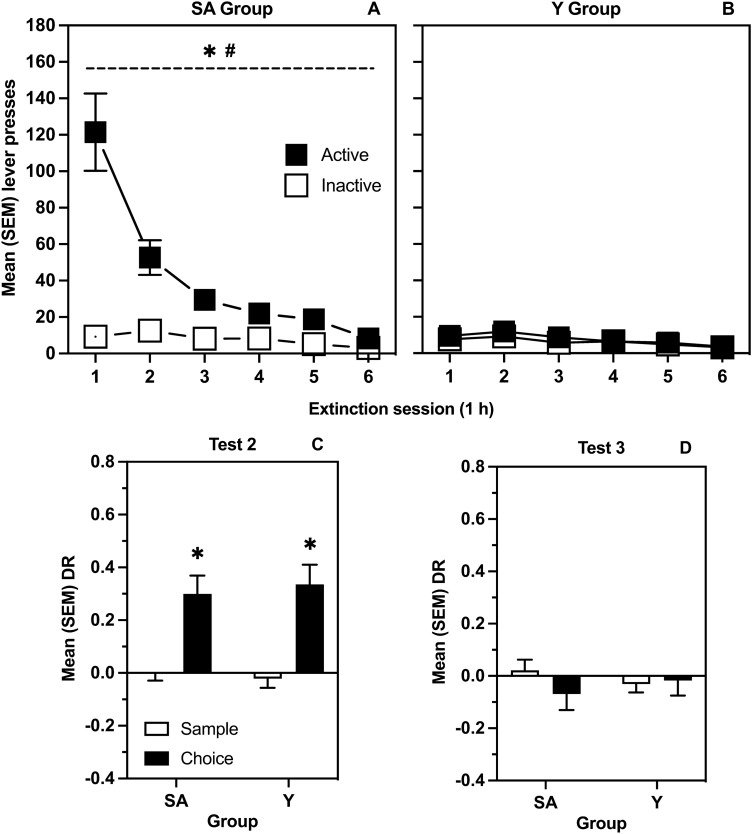
Effects of exposure to contextual heroin CSs on lever responses and object location memory. (**A**) mean (SEM) active and inactive lever presses for rats in the SA group during extinction. (**B**) mean (SEM) active and inactive lever presses for rats in the yoked group during extinction. The * indicates a significant difference between levers. The # indicates a significant difference in responding between groups on the active lever. (**C**) mean (SEM) DRs from sample and choice phases of SA and Y groups on the test of memory modulation by the first extinction session. (**D**) mean (SEM) DRs from sample and choice phases of SA and Y groups on the test of memory modulation by the last extinction session. The * indicates a significant difference between phases.

Figure 4 represents mean (SEM) responses on the previously active and inactive levers across all 6 extinction sessions for SA (Panel A) and Y (Panel B) groups. The ANOVA on lever presses revealed significant Session by Lever by Group [*F*(5, 170) = 16.8, *p* < 0.001], Session by Group [*F*(5, 170) = 16.0, *p* < 0.001], Lever by Group [*F*(1, 34) = 51.1, *p* < 0.001], and Session by Lever [*F*(1.60, 54.4 GG corrected) = 17.6, *p* < 0.001] interactions, as well as significant main effects of Session [*F*(1.56, 53 GG corrected) = 21.8, *p* < 0.001] and of Lever [*F*(1, 34) = 60.8, *p* < 0.001]. Multiple comparisons revealed that responding on the active lever by the SA group significantly decreased over sessions, but no such extinction curve was observed in the Y group. As well, the SA group displayed significantly more responding on the active lever than the Y group across the entire extinction phase.

Figure 4 also represents mean (SEM) sample and choice DRs for Test 2 (Panel C, performed in conjunction with the first extinction session) and Test 3 (Panel D, performed in conjunction with the last extinction session). On Test 2, t-tests comparing sample and choice DRs were significant in both SA [*t*(17) = − 4.05, *p* < 0.001] and Y [*t*(17) = − 4.59, p < 0.001] groups. On Test 3, no significant differences between sample vs choice DRs were found [*p* = 0.24; *p* = 0.85].

**Figure 5:**
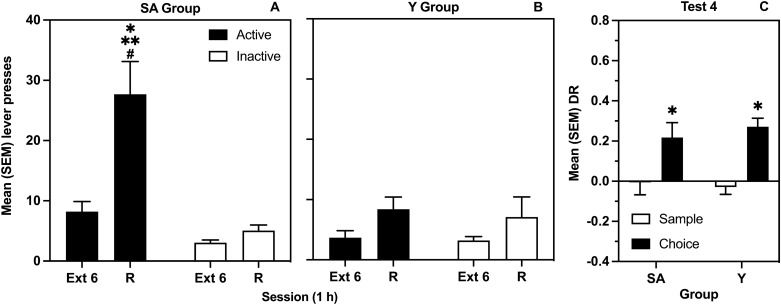
Effects of the discrete heroin CS on reinstatement of lever responses and object location memory. Mean (SEM) active and inactive lever presses during the last extinction (Ext 6) and the cue-induced reinstatement (R) sessions for rats in the SA (**A**) and Y (**B**) groups. The * indicates a significant difference between levers. The ** indicates a significant difference in active lever responding from Ext 6 to R. The # indicates a significant difference in active lever responding between SA and Y groups. (**C**) mean (SEM) DRs from sample and choice phases of SA and Y groups on the test of memory modulation by the reinstatement session. The * indicates a significant difference between phases.

Figure 5 represents mean (SEM) responses of SA (Panel A) and Y (Panel B) groups on the active and inactive levers during the last extinction session (Ext 6) and the reinstatement session (R). The ANOVA revealed significant Session by Lever by Group [*F*(1, 34) = 9.73, *p* = 0.004], Session by Group [*F*(1, 34) = 4.70, *p* = 0.037], Lever by Group [*F*(1, 34) = 12.0, *p* = 0.001] and Session by Lever [*F*(1, 34) = 11.8, *p* = 0.002] interactions, as well as main effects of Session [*F*(1, 34) = 25.8, *p* < 0.001] and of Lever [*F*(1, 34) = 15.4, *p* < 0.001]. Multiple comparisons in the SA group indicated a significant increase in responding selectively on the active lever from Ext 6 to R, and significantly more responding on the active vs inactive lever during reinstatement. In contrast, the increase in responding observed in the Y group during reinstatement was not lever selective, and overall, there was significantly less responding in the Y group in comparison to the SA group.

Figure 5C represents mean (SEM) sample and choice DRs for Test 4 performed in conjunction with the reinstatement session. The t-tests comparing sample and choice DRs were significant in both SA [*t*(17) = − 2.25, *p* = 0.038] and Y [*t*(17) = − 6.70, p < 0.001] groups.

## Discussion

The first objective of the current studies was to explore whether mode of administration would impact the ability of heroin to modulate memory consolidation. The second objective was to examine whether mode of administration could also impact the ability of different drug associated CSs to modulate memory consolidation at various stages of exposure in the absence of heroin. To explore these questions, Study 1 investigated memory modulation by 0, 0.3 and 1 mg/kg heroin injected SC in operant chambers and Study 2 asked a similar question but in rats trained to self-administer 0.05 mg/kg/infusion heroin IV, as well as in rats that received identical amounts of IV heroin but passively, using a yoked design. The post-training effects of injected/self-administered/yoked heroin, extinction and reinstatement sessions on memory consolidation were assessed using the object location memory task. It was found that post-sample heroin enhanced object location memory in injected and yoked, but not self-administering, rats. However, post-sample exposure to the heroin cues (i.e., context or/and light cue) modulated memory equally in all groups. Taken together, these data support the conclusion that mode of administration impacts the cognitive consequences of exposure to drugs but not of environmental stimuli linked to their reinforcing effects.

The current study generated an important and new finding about the effect of heroin on memory consolidation. More specifically, in rats that were able to self-administer heroin (on session 12) following the sample phase of an object location memory task, there was no evidence of memory facilitation when tested 72 h later, drug free, in the choice phase (Fig. 3C). This is notable because animals that received an identical amount of heroin, in the same pattern of intravenous infusions, did display enhanced memory 72 h later (Fig. 3C).

Perhaps, this key difference between self-administered and yoked heroin was observed because drugs administered passively can produce aversive effects^37^. This is a possibility as aversive stimuli, including precipitated morphine withdrawal, can enhance consolidation of object recognition memory^38,39^. However, Study 1 demonstrated that 0.3 and 1 mg/kg heroin passively administered in the same operant chambers enhanced memory at 72 h (Fig. 2A), and similar doses/protocols employed in other conditioning studies revealed clear preferences, not avoidances, for environments associated with the effects of passively injected heroin^32,40^. Furthermore, the overall amounts of heroin administered post-sample in Study 1 and Study 2 were similar: 1 mg/kg SC and an average of 1.48 mg/kg IV. Because there are no obvious reasons to suspect that route of administration could reverse the emotional valence of a given drug, and because there is substantial evidence that post-training administration of opiates can enhance memory consolidation in other tasks and species^8,10,41,42^, it seems more likely that the memory effects observed in the SA and Y groups were caused by factors other than potential aversive effects of heroin.

At this time, exactly what these factors may be remains elusive, but it is possible to postulate a role of prediction error and associated DA activation. In fact, a prediction error is generated when there is a discrepancy between what occurs and what is expected^43–45^ and the neurobiological correlate of this cognitive response is activation of mesolimbic DA^27,46–48^. Accordingly, it is possible that once drug-taking behaviour becomes well established and the outcome of lever pressing is anticipated by the subject, the experience of engaging in those behaviours and the pharmacological effects of the drug become expected, and the DA signal is reduced/lost. Such interpretation is consistent with measures of DA concentrations in the NAc indicating that both passive^18,49,50^ and self-administered^51^ morphine/heroin elevate DA, but that this DA response is lost after repeated heroin self-administration^18^. Because DA plays a key role in memory consolidation^52–54^, and our laboratory has demonstrated that DA antagonists can block the facilitation of memory consolidation by cocaine and nicotine when passively administered^55^, it is possible that self-administered heroin in well trained rats can no longer modulate memory consolidation. Clearly, it will be important to directly explore whether the memory enhancing function of post-sample passive heroin is dependent on DA activity.

The current study also generated three significant findings relevant to the concept of conditioned memory modulation. First, as observed in Study 1 and replicated in Study 2, the context CS in the absence of heroin significantly improved object location memory when experienced post-sample (Figs. 2B, and 4C, respectively). These results are not only consistent with previous findings employing the object recognition task^10^, but also generalize the effect of a drug-paired CS to a task that presumably engages different memory systems^31,56^. Moreover, the fact that this finding was identical in self-administering and yoked groups (Fig. 4C) clearly suggests that the mode of administration plays a minor role in conditioned memory modulation presumably because Pavlovian conditioning occurs regardless of whether a drug is self-administered or passively received.

Second, as observed in Study 1 and replicated in Study 2, the enhancing effect of the context CS on consolidation of object location memory was lost after repeated exposure to the context in the absence of heroin (Fig. 2C, and 4D, respectively), and this occurred regardless of mode and route of administration. As far as we know, this is the first demonstration that conditioned memory modulation follows the Pavlovian principle of extinction^57^, similarly to other conditioned responses. This finding opens a variety of related and interesting questions including whether conditioned memory modulation would also display spontaneous recovery^58^ and/or renewal^59^.

Third, Study 2 indicated that the cue paired with each IV heroin infusion also gained the ability to modulate object location memory consolidation. This observation in the Y group is a further replication the context CS effect outlined above, with the only difference that in this case, this was a discrete CS rather than a context CS. However, this finding is much more interesting in the self-administration group because it co-occurred with reinstatement of extinguished lever pressing. That is, in this group, response-contingent presentation of the discrete CS significantly and selectively increased responding on the previously active lever (Fig. 5A). This typical demonstration of cue-induced reinstatement^15,35^ clearly indicates that the discrete CS was effective in acting as a conditioned reinforcer^60^ and this result represents, as far as we know, the first evidence in support of the “memory enhancing function of reinforcers” hypothesis^3^, applied to conditioned reinforcing stimuli.

In sum, this study examined the potential role for mode of drug administration in the effects of heroin and heroin-paired cues on memory consolidation. Admittedly, object location memory could have been tested at other stages of drug exposure, other memory tasks could have been employed revealing different opiate effects on memory consolidation^61–65^, different heroin doses or schedules could have been used, and the study could have also included female animals as females are known to acquire opiate self-administration quicker, consume more opioids, and be more motivated to consume opioids^66–68^. Notwithstanding these limitations, our data reveal interesting interactions between the pharmacological effect of heroin and environmental cues in determining their shared effects on cognitive processes. That is, heroin’s action on memory formation may be dependent on how the drug is received and may change over repeated administration. Moreover, our data also clearly indicate that a significant level of conditioning occurs to a variety of cues present during drug exposure regardless of how the drug is administered, and these cues appear to have reliable and predictable effects on memory consolidation processes.

## Data Availability

The datasets generated during and/or analysed during the current study are available from the corresponding author upon reasonable request.
